# 4361 Interim Results of the Effect of Haptoglobin Phenotype on Inflammatory Cytokine Concentrations in Plasma and CSF After Aneurysmal Subarachnoid Hemorrhage

**DOI:** 10.1017/cts.2020.72

**Published:** 2020-07-29

**Authors:** Austin Smith

**Affiliations:** 1 University of Kentucky Center for Clinical and Translational Science

## Abstract

OBJECTIVES/GOALS: Haptoglobin (Hp) phenotypes may affect inflammatory response after neurologic injury. We are investigating the relationship between patient Hp phenotypes and inflammatory cytokine concentrations in plasma and CSF after aneurysmal subarachnoid hemorrhage (aSAH), a severe form of hemorrhagic stroke. METHODS/STUDY POPULATION: Following IRB approval, all patients with angiographically-proven aSAH and who underwent extraventricular drain (EVD) placement were included. Patients were excluded if they were not expected to survive hospitalization or were on pre-existing anti-inflammatory agents. For all enrolled patients, plasma and CSF samples were taken on post-bleed days 3, 5, 7, and 10, processed and then frozen for later analysis. In this interim analysis, Hp phenotype was assessed through ELISA analysis of plasma samples and cytokine concentrations were determined using multiplex ELISA kits for both plasma and CSF samples. Hp phenotypes were dichotomized to either HP1 (Hp 1-1 or 1-2) or HP2 (Hp 2-2). RESULTS/ANTICIPATED RESULTS: To date, 23 aSAH patients have been enrolled in this IRB-approved study. An interim analysis of the first 13 patients has revealed eight Hp1 patients (Hp 1-1 n = 1, Hp 1-2 n = 7) and five Hp2 patients. CSF levels of IL-6 and TNF-a were greater than plasma levels in all patients at all time points. CSF levels of IL-6 appear to peak on PBD 5 (1890 ± 767 pcg/mL) and 7 (1612 ± 899 pcg/mL). The CSF IL-6 concentrations in the Hp2 group were lower (1792 ± 806 pcg/mL vs. 1952 ± 791 pcg/mL) on PBD5 but were higher (1635 ± 930 pcg/mL vs 1598 ± 943 pcg/mL) at PBD7; however, these differences did not reach statistical significance. DISCUSSION/SIGNIFICANCE OF IMPACT: This interim analysis demonstrated no statistically significant differences in plasma or CSF cytokine concentrations between patients with different Hp phenotypes. This may be due to the low number of samples or the potential confounding effect of disease-specific secondary neurological injuries.

